# MicroRNA-545 Suppresses Cell Proliferation by Targeting Cyclin D1 and CDK4 in Lung Cancer Cells

**DOI:** 10.1371/journal.pone.0088022

**Published:** 2014-02-05

**Authors:** Bowen Du, Zhe Wang, Xin Zhang, Shipeng Feng, Guoxin Wang, Jianxing He, Biliang Zhang

**Affiliations:** 1 The State Key Laboratory of Respiratory Diseases, Guangzhou Institutes of Biomedicine and Health, Chinese Academy of Sciences, Guangzhou, China; 2 School of Life Sciences, University of Science and Technology of China, Hefei, China; 3 The State Key Laboratory of Respiratory Diseases, Department of Cardiothoracic Surgery, The First Affiliated Hospital of Guangzhou Medical University, Guangzhou, China; 4 Guangzhou RiboBioCo, Ltd., Guangzhou, China; Sun Yat-sen University, China

## Abstract

An increasing number of reports have shown that diverse microRNAs are involved in tumorigenesis and tumor progression. We performed this study to identify novel miRNAs that may be involved in lung cancer and study on their functions. We tested the expression of 450 miRNAs in lung tumor tissues and adjacent non-cancerous tissues. We found that miRNA-545 was less abundant in cancerous lung tissues than in adjacent non-cancerous tissues. Our further studies showed that miR-545 suppressed cell proliferation *in vitro* and *in vivo*. We also found that miR-545 caused cell cycle arrest at the G0/G1 phase and induced cell apoptosis in lung cancer cells by targeting cyclin D1 and CDK4 genes. The effects of cyclin D1 and CDK4 down-regulated by miR-545 were similar to those caused by siRNAs of cyclin D1 and CDK4, and overexpression of cyclin D1 and CDK4 could abolish the miR-545-induced inhibition of cell proliferation. In conclusion, miR-545 suppressed cell proliferation by inhibiting the expression of cyclin D1 and CDK4. Our findings provide new knowledge regarding the role of miR-545 in the development of lung cancer and indicate the potential application of miR-545 in cancer therapy.

## Introduction

MicroRNAs (miRNAs) are a class of small noncoding RNAs that are approximately 22 nucleotides in length [1], [2], and miRNAs can suppress posttranscriptional gene expression by binding to the 3′-untranslated regions (3′UTRs) of mRNAs, which induces translational inhibition or target mRNA degradation [3].

Cyclin D1 and CDK4 work together in the same complex to regulate the cell cycle G1/S transition. Previous studies have shown that cyclin D1 and CDK4 are more abundant in lung tumor tissues than in normal lung tissues [4], [5]. An increasing body of evidence supports a central role for cyclin D1 and CDK4 in promoting cancer cell proliferation. Oliver et al. reported cyclin D1 as a “pivotal element” of malignant transformation in lung cancer [6]. Accordingly, silencing cyclin D1 with antisense oligonucleotides can inhibit the proliferation of non-small cell lung cancer cells [7]. These studies indicate that cyclin D1 and CDK4 may be important genes for lung cancer cell proliferation.

miRNAs are involved in cell proliferation [8], differentiation [9], and apoptosis [10], and aberrant miRNA expression is linked to tumor formation and progression [11]–[14]. Many studies have shown that miRNAs can function as oncogenes or tumor suppressor genes in lung cancer. For example, the miR-17-92 cluster is overexpressed in lung cancer cells and promotes cell proliferation in a lung cancer cell line [15]. Moreover, antisense oligonucleotides complementary to miR-17-5p and miR-20a, which are members of the miR-17-92 cluster, can induce cell apoptosis [16]. However, let-7 miRNA functions as a tumor suppressor gene in lung cancer and is less abundant in lung cancer cells than in normal lung cells [17]. Overexpression of let-7 suppresses cell growth in multiple lung cancer cell lines and xenografts in immunodeficient mice [18]. miR-34a is another important tumor suppressor miRNA. miR-34a is underexpressed in primary non-small cell lung cancer (NSCLC) tumor tissues compared with paired normal tissues [19]. Additionally miR-34a can inhibit NSCLC cell proliferation [20]. miR-210 is overexpressed at late stages of NSCLC and involved in alteration of cell viability in the lung adenocarcinoma A549 cell line [21]. miR-23 level is elevated in the sera from NSCLC patients compared with those from healthy subjects [22]. These reports reveal that various miRNAs are involved in the regulation of lung cancer.

The goal of this study was to identify novel miRNAs associated with lung cancer and to elucidate their functions. We utilized quantitative real-time polymerase chain reaction (qRT-PCR) assays to study the miRNA profile in lung cancer, and the results revealed that miR-545 was underexpressed in lung tumor tissues compared with adjacent non-cancerous lung tissues. We found that miR-545 suppresses cell proliferation by directly targeting cyclin D1 and CDK4 genes in lung cancer cell lines. Our results indicate that miR-545 functions as a tumor suppressor in lung cancer.

## Results

### miR-545 is Decreased in Human Lung Cancer Tissues

We compared the expression of 450 miRNAs between lung cancer tissues and adjacent non-cancerous lung tissues from 15 patients, and we found that 31 miRNAs were underexpressed (P<0.05, fold change <0.5) in lung cancer tissues compared with adjacent non-cancerous lung tissues (Fig. S1,Table S1 and S2).

**Figure 1:**
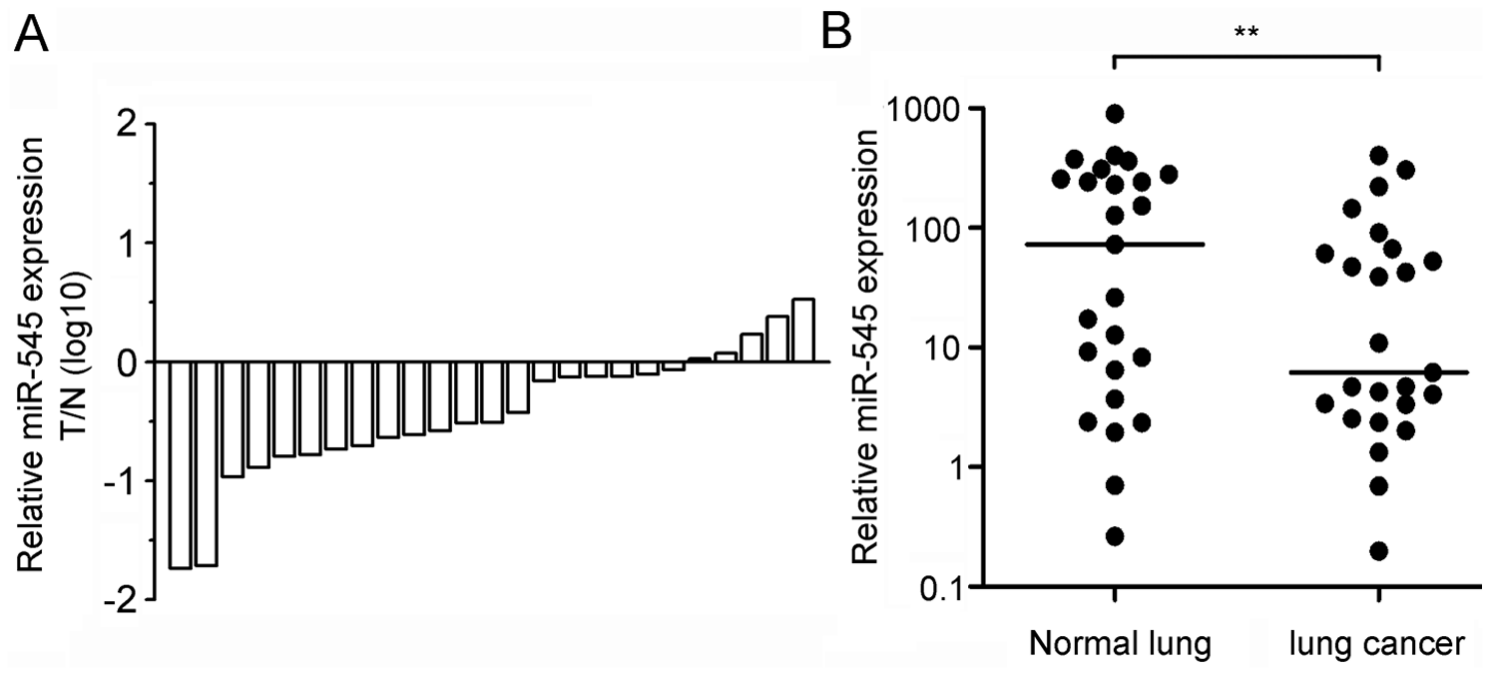
miR-545 is underexpressed in lung cancer tissues. (a) Relative miR-545 expression in 25 paired lung cancer tumor tissues and adjacent non-cancerous tissues. (b) Expression of miR-545 in lung cancer tissues compared with adjacent non-cancerous tissues. 5S rRNA was used as an internal control. The data were analyzed using the 2^−ΔΔCt^ method. *, P<0.05 (Wilcoxon test).

To confirm the expression of miR-545, we tested miR-545 expression in another 10 patients. We found that miR-545 levels in lung cancer tissues were less than 50% of those in adjacent non-cancerous lung tissues in 14 of the 25 patients, whereas miR-545 levels in lung cancer tissues were more than twice those in adjacent non-cancerous lung tissues in 2 of the 25 patients (Fig. 1A). The analysis of multiple sets of comparable cancerous and adjacent non-cancerous lung tissues indicated that miR-545 was less abundant in lung cancer tissues than in adjacent non-cancerous tissues (Fig. 1B). This suggests that miR-545 was underexpressed in lung tumors and might function as a tumor suppressor.

### miR-545 Inhibits Cell Proliferation in vitro and in vivo

**Figure 2:**
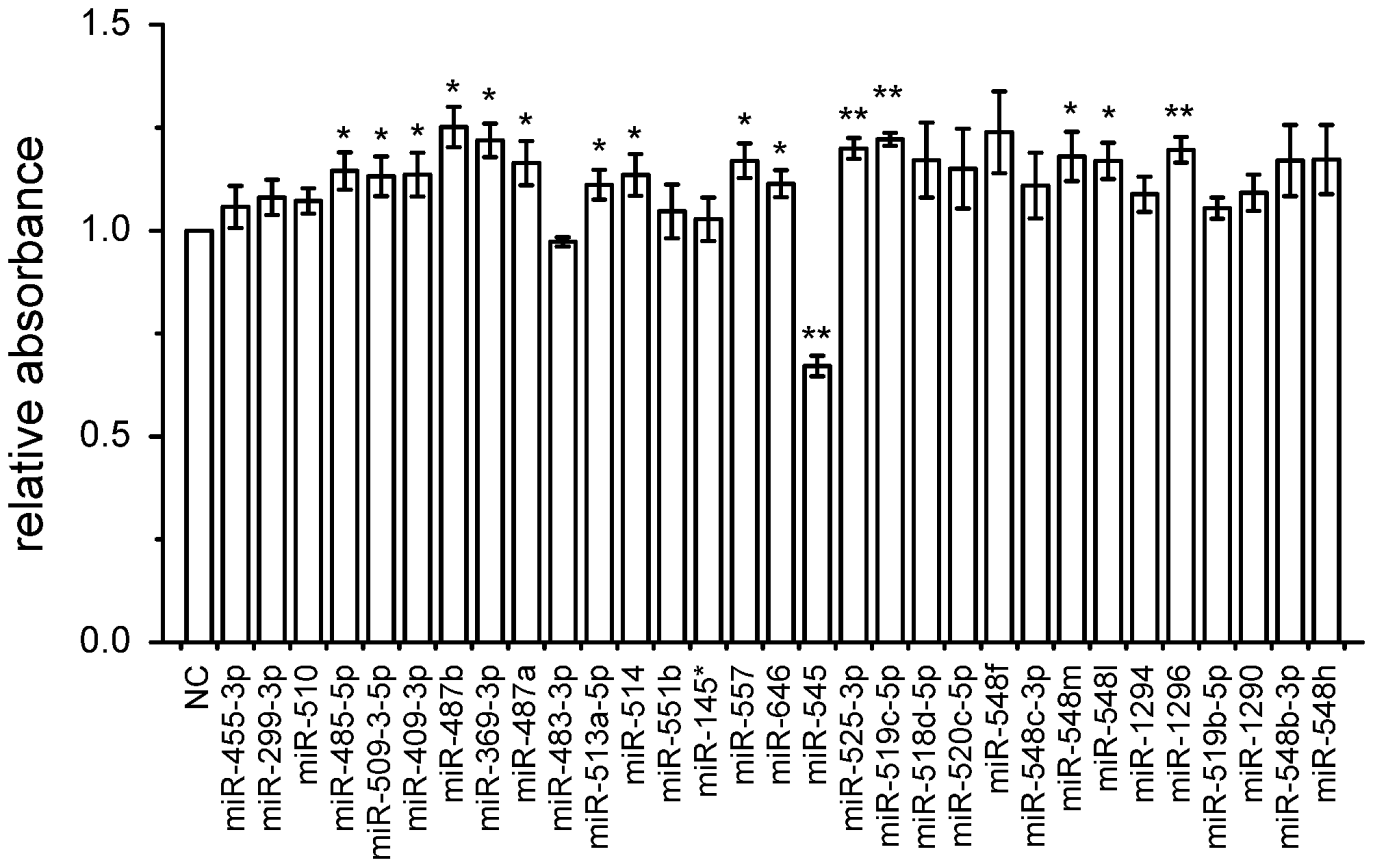
Proliferation assay of A549 cells transfected with the selected miRNAs. A549 cell viability was measured by CCK-8 at 72 h after transfection with each miRNA. The data are presented as the mean ± S.D. from three independent experiments. NC, no-target control; *, P<0.05; **, P<0.01 (Student’s t test).

To identify miRNAs that might be involved in cell proliferation, we used the CCK-8 kit to study the viability of A549 cells transfected with each of the 31 miRNAs. We found that A549 cells transfected with miR-545 had the lowest cell viability (Fig. 2).

**Figure 3:**
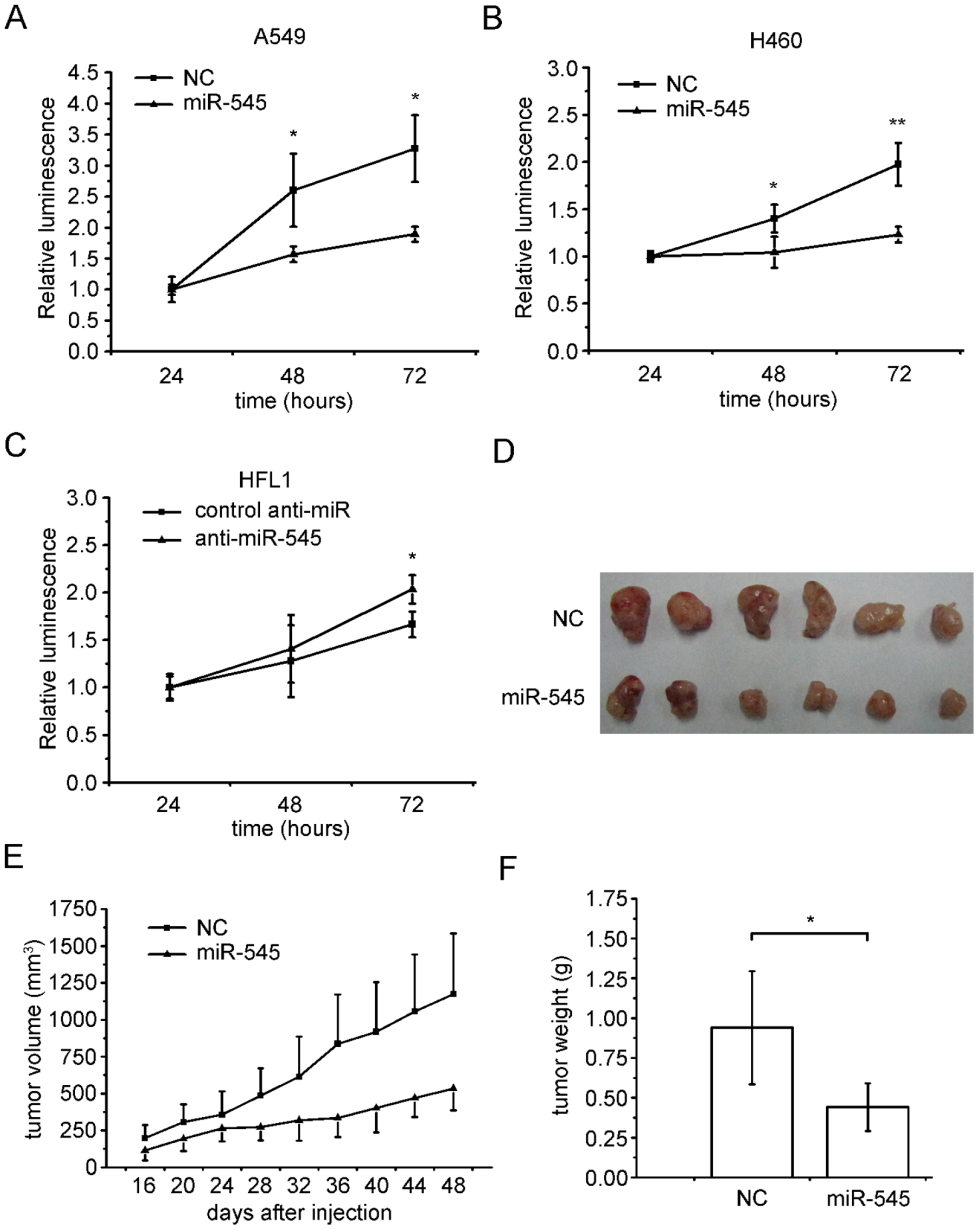
miR-545 suppresses cell proliferation *in vitro* and *in vivo*. Transfection with miR-545 mimics suppresses the viability of (a) A549 cells and (b) H460 cells. (c) Transfection with an miR-545 inhibitor increases the viability of HFL1 cells. (d) Tumor images from the nude mouse xenograft model. (e) Volumes and (f) weights of tumors in a nude mouse xenograft model. The data are presented as the means ± S.D. from three independent experiments. *, P<0.05; **, P<0.01 (Student’s t test).

To confirm that miR-545 can suppress cell proliferation, we performed the cell viability assay with three cell lines. A549 and H460 cells transfected with miR-545 mimics showed lower cell viability than those transfected with a no-target control (Fig. 3A and 3B); however, HFL1 lung fibroblasts transfected with an miR-545 inhibitor exhibited a more efficient cell proliferation compared with those transfected with a control inhibitor (Fig. 3C).

We next used a tumor xenograft mouse model to test the effect of miR-545 on tumor progression. The A549 cells were pre-treated with miR-545 mimics or a no-target control and were subsequently injected into athymic nude mice. The tumor volume in mice that received cells pre-treated with miR-545 mimics was significantly (P<0.05) smaller than that in mice that received cells treated with a no-target control. These differences in volume and weight were observed in tumors harvested from mice sacrificed at day 48 (Fig. 3D, 3E, and 3F). The results show that miR-545 can significantly inhibit the lung cancer cell growth in a nude mouse xenograft model.

### miR-545 Induces Cell Cycle Arrest at the G0/G1 Phase

**Figure 4:**
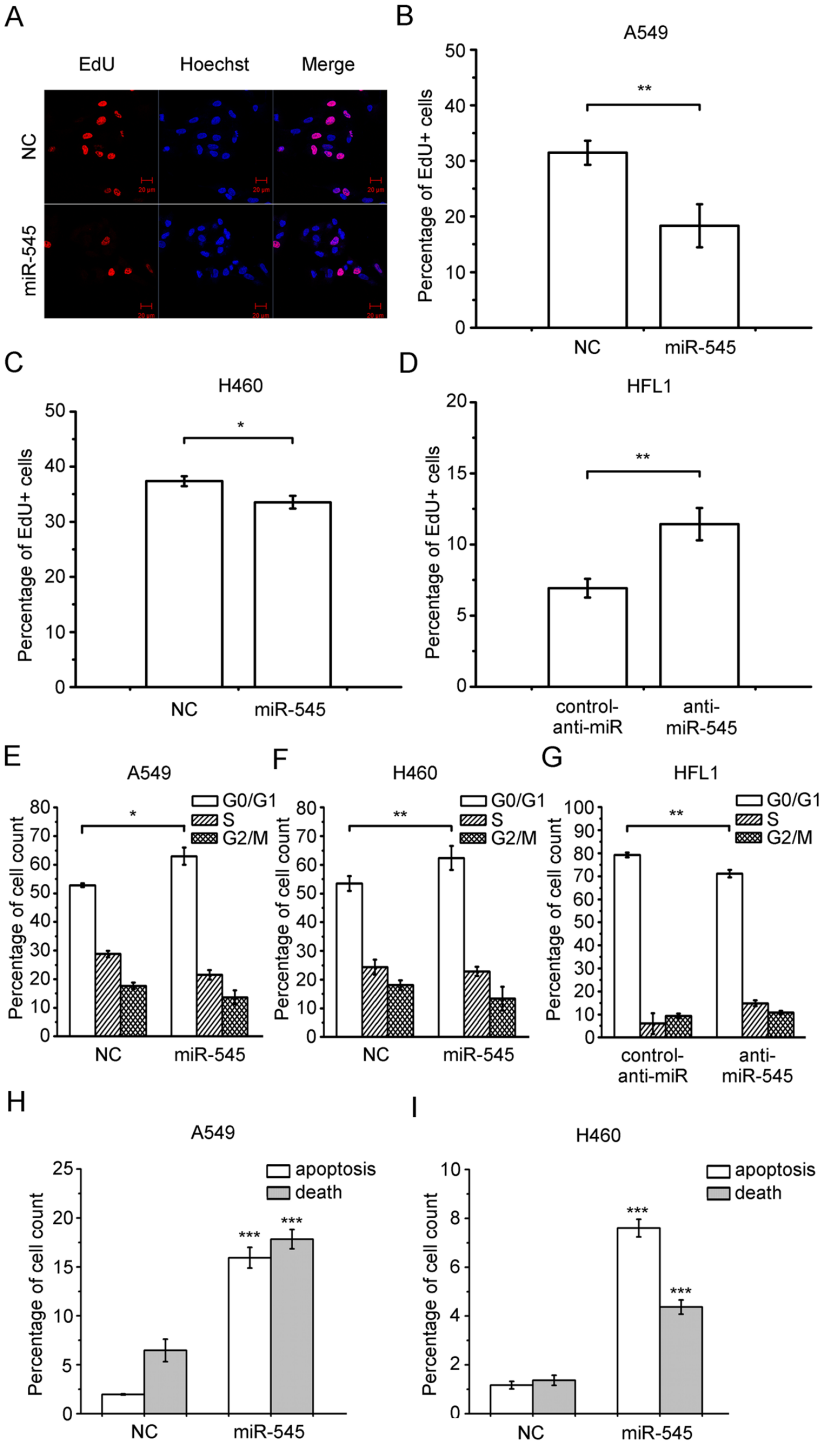
miR-545 inhibits DNA synthesis and causes cell-cycle arrest. (a) EdU incorporation in A549 cells measured by confocal laser microscopy. Transfection with miR-545 inhibits DNA synthesis in (b) A549 cells and (c) H460 cells. (d) Transfection of HFL1 cells with miR-545 inhibitor increases DNA synthesis. Transfection with miR-545 increases the percentage of cells in the G0/G1 phase in (e) A549 cells and (f) H460 cells (g) Transfection of HFL1 cells with miR-545 reduces the percentage of cells in the G0/G1 phase. miR-545 induces apoptosis in (h) A549 and (i) H460 cells. Data are presented as the mean ± S.D. from three independent experiments. NC, no-target miRNA control; *, P<0.05; **, P<0.01 (Student’s t test).

An EdU assay revealed that both A549 and H460 cells transfected with miR-545 mimics incorporated less EdU than those transfected with a no-target control (Fig. 4A–C). In contrast, HFL1 cells transfected with the miR-545 inhibitor incorporated more EdU than those transfected with the control inhibitor (Fig. 4D). These results suggest that miR-545 may inhibit DNA synthesis. To further probe the regulatory mechanism(s) of miR-545, we conducted a cell-cycle assay. A higher proportion of A549 and H460 transfected with miR-545 mimics were in the G0/G1 phase compared with those transfected with a no-target control (Fig. 4E and 4F). In contrast, a smaller proportion of HFL1 cells transfected with an miR-545 inhibitor were in the G0/G1 phase compared with those transfected with a control anti-miRNA inhibitor (Fig. 4G). These results demonstrate that miR-545 arrests the cell cycle in the G0/G1 phase, thus inhibiting DNA synthesis and cell proliferation. Moreover, we found that miR-545 induced cell apoptosis and death in A549 and H460 cells (Fig. 4H and 4I).

### miR-545 Directly Targets Cyclin D1 and CDK4

**Figure 5:**
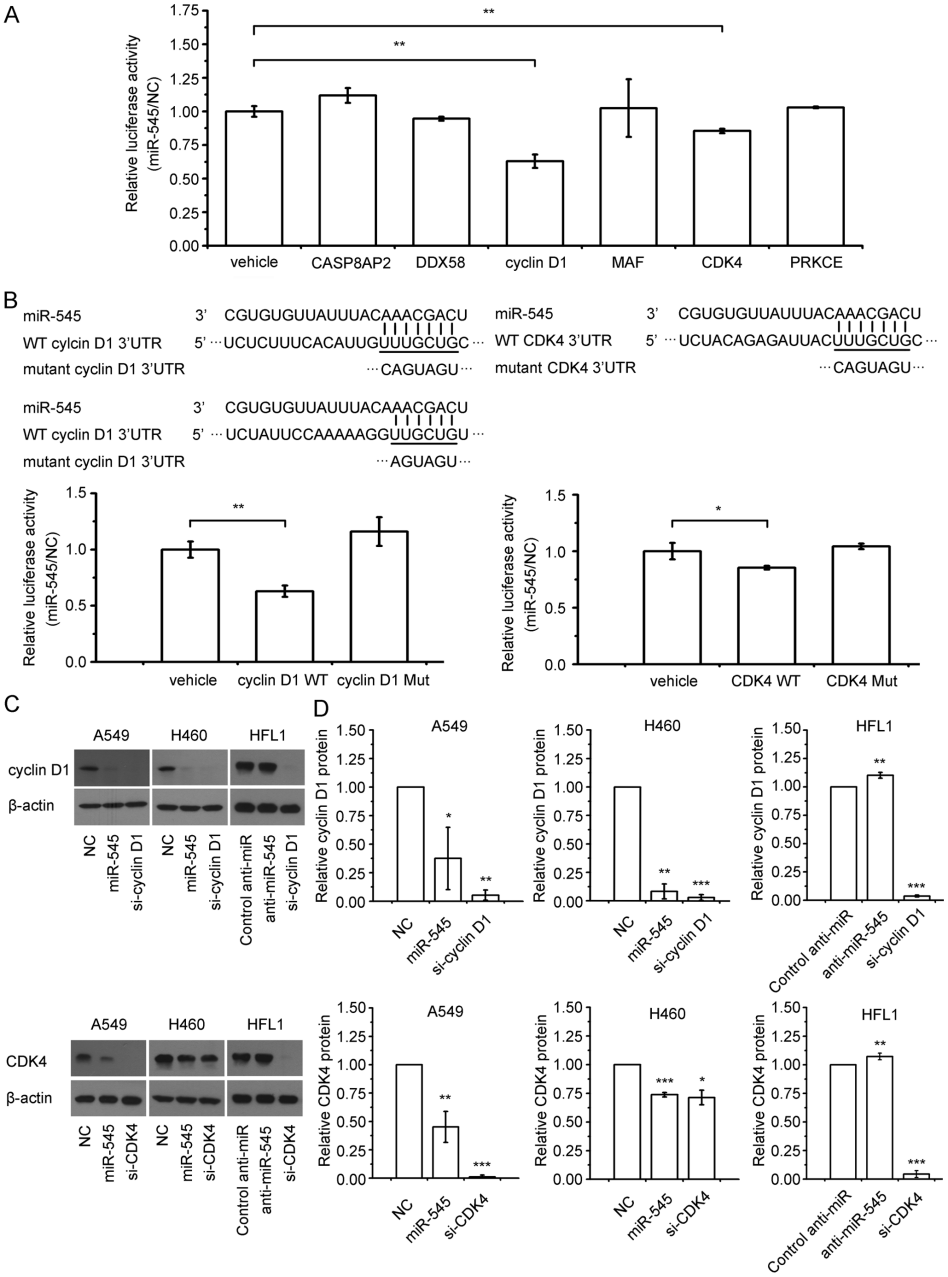
miR-545 targets cyclin D1 and CDK4 genes. (a) miR-545 inhibited the activity of luciferase containing the 3′UTR of the cyclin D1 or CDK4 gene. pmirGlo plasmids containing wild-type (WT) 3′UTRs of the CASP8AP2, DDX58, cyclin D1, MAF, CDK4, or PRKCE gene were co-transfected into A549 cells with the control miRNA or miR-545. Luciferase activity was measured 48 h after transfection. The normalized ratio of luciferase activity was calculated as (Rluc_miR-545_/Luc_miR-545_)/(Rluc_NC_/Luc_NC_). (b) miR-545 did not inhibit the activity of luciferase constructs containing the mutated (Mut) 3′UTR of the cyclin D1 or CDK4 gene. (c) The abundance of cyclin D1 and CDK4 proteins were analyzed in A549, H460, and HFL1 cells by western blotting after transfection treatments. (d) Levels of cyclin D1 and CDK4 proteins were quantified using ImageJ software. β-actin was used as an internal control. The data are presented as the mean ± S.D. from three independent experiments. *, P<0.05; **, P<0.01; ***, P<0.001 (Student’s t test).

We used the Targetscan software to predict the possible targets of miR-545. Among hundreds of predicted targets, we chose CASP8AP2, DDX58, cyclin D1, MAF, CDK4, and PRKCE as the putative targets because they might be involved in the cell cycle or apoptosis. We cloned the 3′UTRs of these genes into the pmirGlo plasmid and examined whether miR-545 can suppress the expression of these genes by the luciferase assay. The luciferase assay showed that miR-545 mimics inhibited the activity of two reporter constructs that contained the 3′UTR of either cyclin D1 or CDK4 (Fig. 5A). Furthermore, to test whether cyclin D1 and CDK4 are direct targets of miR-545, we mutated the predicted binding site of miR-545 in the 3′UTRs of both genes. We found that miR-545 did not inhibit the activity of reporter constructs that contained the mutant 3′UTRs (Fig. 5B).

We also examined the expression of the endogenous cyclin D1 and CDK4 genes at both the mRNA and protein levels. We found that miR-545 reduced the level of CDK4 mRNA in A549 cells but did not affect the level of cyclin D1 mRNA (Fig. S2). However, we observed that A549 and H460 cells transfected with miR-545 had lower levels of both cyclin D1 and CDK4 proteins than those transfected with a no-target control (Fig. 5C and 5D). Furthermore, HFL1 cells transfected with miR-545 inhibitor had higher levels of both cyclin D1 and CDK4 proteins than those transfected with NC inhibitors (Fig. 5C and 5D).

### miR-545 Suppresses Tumor Proliferation by Targeting Cyclin D1 and CDK4

**Figure 6:**
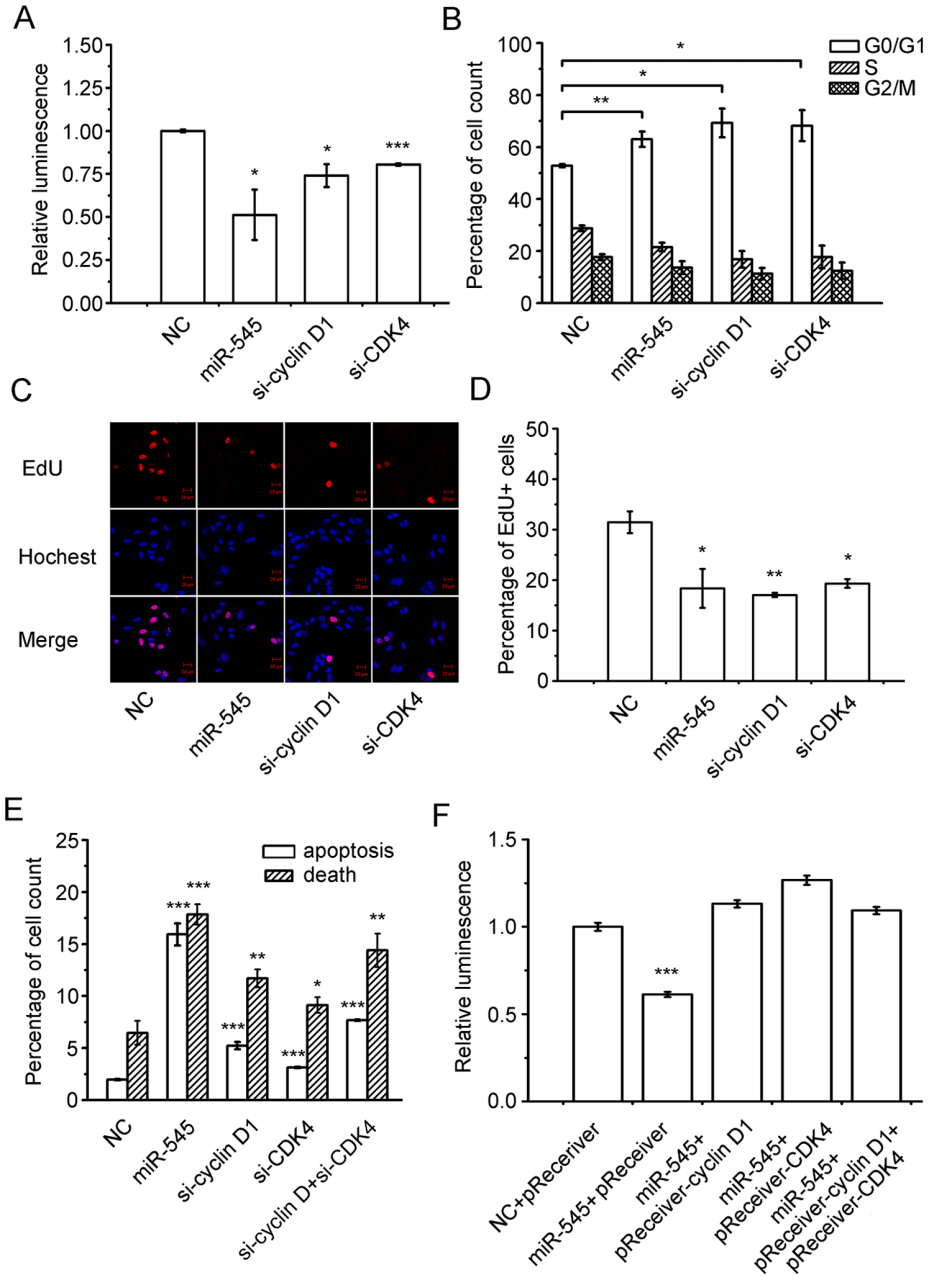
miR-545 and siRNAs target cyclin D1 and CDK4 genes to suppress cell proliferation. (a) miR-545 mimics and siRNAs inhibited A549 cell viability at 72 h after transfection. (b) The cell-cycle arrested at G0/G1 phase after transfection of A549 cells with miR-545 and siRNAs targeting cyclin D1 and CDK4. miR-545 and siRNA induced inhibition of DNA synthesis as determined by the EdU incorporation assay using (c) confocal laser microscopy and (d) flow cytometry. (e) Underexpression of cyclin D1 and CDK4 induces cell apoptosis in A549 cells. (f) Overexpression of cyclin D1 and CDK4 abolishes the inhibition caused by miR-545. The data are presented as the mean ± S.D. from three independent experiments. *, P<0.05; **, P<0.01; ***, P<0.001. Student’s t test was used for (a)–(e); one-way ANOVA was used for (f).

To examine whether miR-545 inhibits cell viability by targeting cyclin D1 and CDK4, we used siRNAs to inhibit the expression of cyclin D1 and CDK4 genes; the results were similar to those described above for miR-545. For example, the use of siRNAs to silence either cyclin D1 or CDK4 reduced the viability of A549 cells (Fig. 6A). The siRNA-mediated suppression of cyclin D1 and CDK4 genes caused cell cycle arrest at the G0/G1 phase (Fig. 6B), inhibited EdU incorporation (Fig. 6C and 6D) and induced apoptosis in A549 cells (Fig. 6E). Furthermore, the overexpression of cyclin D1 and/or CDK4 genes could significantly abrogate miR-545-induced inhibition of A549 cell growth (Fig. 6F). Taken together, these results reveal that the cyclin D1 and CDK4 genes are direct targets of miR-545.

## Discussion

Our study shows that miR-545 is underexpressed in lung cancer tumors, and miR-545 has been reported to be underexpressed in gastric carcinoma [23]. Furthermore, miR-545 inhibits the proliferation of lung cancer cells both *in vitro* and *in vivo*. These results indicate miR-545 might function as a tumor suppressor in lung cancer.

Further investigation reveals that cyclin D1 and CDK4 genes are direct targets of miR-545. miR-545 inhibits cyclin D1 and CDK4 gene expression by recognizing sequences in their 3′UTRs. Furthermore, overexpression of cyclin D1 and CDK4 can abolish the inhibition of proliferation caused by miR-545 mimics. These data suggest that miR-545 inhibits cell proliferation by directly targeting cyclin D1 and CDK4. Previous studies report that the expression of cyclin D1 and CDK4 in human lung cancer tissues is substantially higher than that in normal lung tissues [7], [8]. Our results indicate that overexpression of cyclin D1 and CDK4 in lung cancer tissues may partially result from the underexpression of miR-545.

In conclusion, the miRNA profiling results show that miR-545 is less abundant in human lung cancer tissues than in adjacent non-cancerous lung tissues. miR-545 inhibits the proliferation of lung cancer cells by repressing the expression of cyclin D1 and CDK4 genes. An enhanced understanding of miR-545 dysregulation in lung cancer cells contributes to our understanding of the molecular mechanisms responsible for lung cancer. Moreover, miR-545 might be a potential target for lung cancer therapeutic treatment.

## Materials and Methods

### Ethics Statement

We obtained written informed consent from all patients, and all protocols were approved by the Institutional Review Board of the First Affiliated Hospital of Guangzhou Medical College. All human materials were used in accordance with the policies of the Institutional Review Board. The animal study proposal was approved by the Institutional Animal Care and Use Committee of Guangzhou Institutes of Biomedicine and Health (IACUC 2012010). All experimental procedures involving animals were performed in accordance with the Guide for the Care and Use of Laboratory Animals and were performed according to the institutional ethical guidelines for animal experiments.

### Cell Lines

The human lung cancer cell lines A549 (ATCC) and NCI-H460 (H460, ATCC) were obtained from Dr. Duanqing Pei’s Lab at the Guangzhou Institutes of Biomedicine and Health and were cultured in RPMI-1640 medium (1640, GIBCO, NY, USA) supplemented with 10% fetal bovine serum (FBS, Hyclone, UT, USA). The normal lung fibroblast cell line HFL1 was purchased from the Chinese Academy of Sciences Cell Bank of Type Culture Collection (CBTCCCAS, Shanghai, China) and was cultured in F-12K medium (Jinuo, Hangzhou, China) supplemented with 10% FBS. All cells were cultured in a humidified atmosphere containing 5% CO_2_ at 37°C.

### Tissue Samples Obtained from Lung Cancer Patients

Both cancerous and adjacent non-cancerous lung tissue samples were obtained from the Guangzhou Institute of Respiratory Disease, which is associated with the First Affiliated Hospital of Guangzhou Medical University (Guangzhou, China). Surgically removed samples were stored in liquid nitrogen until use. The relevant characteristics of the studied subjects are shown in Table S3.

### Transfection

All siRNAs, miRNA mimics, and miRNA inhibitors were purchased from Guangzhou RiboBio (RiboBio, Guangzhou, China) and transfected into cells using Lipofectamine 2000 (Invitrogen, Life Technologies, CA, USA) as recommended by the manufacturer. Cells were transfected with miRNA mimics and inhibitors at a concentration of 50 nM. The sequences of siRNAs are as follows: si-cyclin D1 sense, 5′-UGG AAU AGC UUC UGG AAU UdTdT-3′, antisense, 3′-dTdAA CCU UAU CGA AGA CCU UAA-5′; si-CDK4 sense, 5′-CAG CCG AAA CGA UCA AGG AdTdT-3′, antisense, 3′-dTdTG UCG GCU UUG CUA GUU CCU-5′.

### Cell Proliferation Assay

Cell proliferation was measured using Cell Counting Kit 8 reagent (CCK-8, DOJINDO, Kyushu, Japan) or CellTiter-Glo reagent (Promega, WI, USA) according to the manufacturer's instructions.

### EdU Cell Proliferation Assay

Incorporation of the thymidine analog 5-ethynyl-2′-deoxyuridine (EdU) into DNA during DNA replication can be used to measure cells undergoing DNA replication. The percentage of cells incorporating EdU was evaluated using the Cell-Light EdU imaging detecting kit following the manufacturer’s instructions (RiboBio, Guangzhou, China).

### Cell Cycle Assay

Cells were harvested 72 h after transfection and fixed overnight in 70% ice-cold ethanol. After fixation, the cells were treated with 500 ng/µl of RNase A for 30 min and subsequently stained with 50 ng/µl of propidium iodide (PI) for 20 min. The cells were quantified using fluorescence activated cell sorting (FACS, BD, NJ, USA), and the data were analyzed by FlowJo software (Tree Star, OR, USA).

### Cell Apoptosis Assay

Cell apoptosis analysis was performed with the PE Annexin V Apoptosis Detection Kit I (BD, NJ, USA) according to the manufacturer’s protocol, and the cells were analyzed using FACS (BD, NJ, USA).

### RNA Extraction and qRT-PCR

Total RNA was extracted from the cell lines and tissue samples using Trizol reagent (Invitrogen, Life Technologies, CA, USA) according to the manufacturer’s instructions. Synthesis of cDNA using M-MLV Reverse Transcriptase (Promega, WI, USA) was performed as suggested by the manufacturer, and random primers (Takara, Shiga, Japan) were used for mRNA. The qRT-PCR assays were performed using the SYBR Green RealMasterMix kit (Tiangen, Beijing, China) according to the manufacturer’s instructions. Reverse Transcription and qRT-PCR for miRNA were performed using the Bulge-Loop miRNA qRT-PCR Primer Set (RiboBio, Guangzhou, China). Data analysis was performed using the 2^−ΔΔCt^ method [24]. The following primers were used for the analysis: cyclin D1 forward primer, 5′-CAA ATG GAG CTG CTC CTG GTG-3′, reverse primer, 5′-CTT CGA TCT GCT CCT GGC AGG-3′; CDK4 forward primer, 5′-TGC CAA TTG CAT CGT TCA CCG AG-3′, reverse primer, 5′-TGC CCA ACT GGT CGG CTT CA-3′; actin forward primer, 5′-CTC CAT CCT GGC CTC GCT GT-3′, reverse primer, 5′-GCT GTC ACC TTC ACC GTT CC-3′; and 5S rRNA forward primer, 5′-ACG GCC ATA CCA CCC TGA AC-3′, reverse primer, 5′-GGC GGT CTC CCA TCC AAG TA-3′.

### Plasmid Construction

The 3′UTRs of cyclin D1 and CDK4 genes were cloned into the pmirGlo plasmid (Promega, WI, USA) between the *Xho*I and *Xba*I sites using the following primers: cyclin D1 wild-type 3′UTR forward primer, 5′-ATA TCT CGA GCA GGT GCT CCC CTG ACA GTC CCT-3′, reverse primer, 5′-TAA TCT AGA TGG AAA CAT GCC GGT TAC ATG TTG GT-3′; CDK4 wild-type 3′UTR forward primer, 5′-ATA TCT CGA GGC AAT GGA GTG GCT GCC ATG GAA-3′, reverse primer, 5′-TAA TCT AGA TTG ACA CAG AGT CTT GCT CTG TTG CCC A-3′.

The pmirGlo plasmids containing mutated cyclin D1 or CDK4 3′UTR were constructed using the KOD-plus mutagenesis kit (Toyobo, Osaka, Japan) according to the manufacturer's instructions.

The open reading frame expression vectors pReceiver_M02_cyclin D1, pReceiver_M02_CDK4, and the control vector pReceiver_M02 were purchased from the FulenGen Corporation (GeneCopoeia, MD, USA). All inserted or mutated sequences were confirmed by sequencing.

### Dual-luciferase Assay

Cells were seeded in 96-well plates 1 day prior to transfection. The miRNA mimics (50 nM) and plasmid (5 ng/µL) were co-transfected into A549 cells. At 48 h after transfection, luciferase activity was measured using the Dual-Glo luciferase assay kit (Promega, WI, USA) according to the manufacturer’s instructions.

### Western Blotting

Cells were harvested and resuspended in SDS buffer (Beyotime, Shanghai, China) for preparation of total protein extracts. Briefly, total protein extract was separated by 12% sodium dodecyl sulfate-polyacrylamide gel electrophoresis and transferred onto an Immobilon-P transfer polyvinylidene fluoride membrane (Millipore, MA, USA). The membrane was incubated at room temperature in 5% bovine serum albumin (BSA) prepared with TBS-T buffer for 2 h. The membrane was incubated with primary antibody diluted 1∶1,000 with 1% BSA at 4°C overnight. On the following day, the membrane was incubated with secondary horseradish peroxidase (HRP)-conjugated antibody diluted 1∶5,000 with 1% BSA at 4°C for 6 h. The membrane was visualized after incubation in HRP substrate (BeyoECL plus A/B, Beyotime, Shanghai, China). The antibodies used in this study were anti-cyclin D1 (sc-8396, Santa Cruz, CA, USA), anti-CDK4 (sc-260, Santa Cruz, CA, USA), anti-β-actin (A2066, Sigma, MO, USA), HRP-conjugated anti-mouse IgG (sc-2005, Santa Cruz, CA, USA), and HRP-conjugated anti-rabbit IgG (sc-2004, Santa Cruz, CA, USA).

### Tumor Xenografts

At 6 h after transfection of miR-545 mimics or control miRNA, A549 cells were harvested and suspended in phosphate-buffered saline at a concentration of 1×10^6^ cells/mL and injected into either side of the posterior flank of the same BALB/c athymic nude mice (5–6 weeks of age) with 100 µL cell suspension. Tumor growth was measured every 4 days. Tumor volume (V) was monitored by measuring the length (L) and width (W) of the tumor with calipers and was calculated using the formula V = (L×W^2^)/2 [25].

### Statistical Analysis

The data are presented as the mean ± standard deviation (S.D.). P<0.05 was considered significant. All statistical analyses were performed using SPSS 16.0 software (Chicago, IL). Student’s t test was used to analyze the significance between two groups. One-way Analysis of Variance (ANOVA) was used to test the significance among more than two groups. The analysis of miRNAs expression in clinical sample was performed using Wilcoxon test.

## Supporting Information

Figure S1 MiRNA expression in lung cancer tissues and adjacent non-cancerous tissues. The expressions of miRNAs are measured with qRT-PCR. 5S rRNA was used as an internal control. The heatmap represents the overexpression (red) and underexpression (green) of miRNAs. Missing data is represented with gray color.
(TIF)

Figure S2 Cyclin D1 and CDK4 mRNAs were measured by qRT-PCR. β-actin was used as an internal control. Data are presented as mean ± S.D. *, P<0.05; **, P<0.01; ***, P<0.001(Student’s t test).
(TIF)

Table S1 The expression of miRNAs in lung cancer tissue and non-cancerous lung tissues.
(DOC)

Table S2 Relative expression of 450 miRNAs.
(XLS)

Table S3 The characteristics of clinical lung cancer patients.
(DOC)
