# Supplementary figures and images for: MicroRNA-545 Suppresses Cell Proliferation by Targeting Cyclin D1 and CDK4 in Lung Cancer Cells
Source: PLoS One. 2014 Feb 5;9(2):e88022. doi: 10.1371/journal.pone.0088022 (PMC3914893; doi:10.1371/journal.pone.0088022)

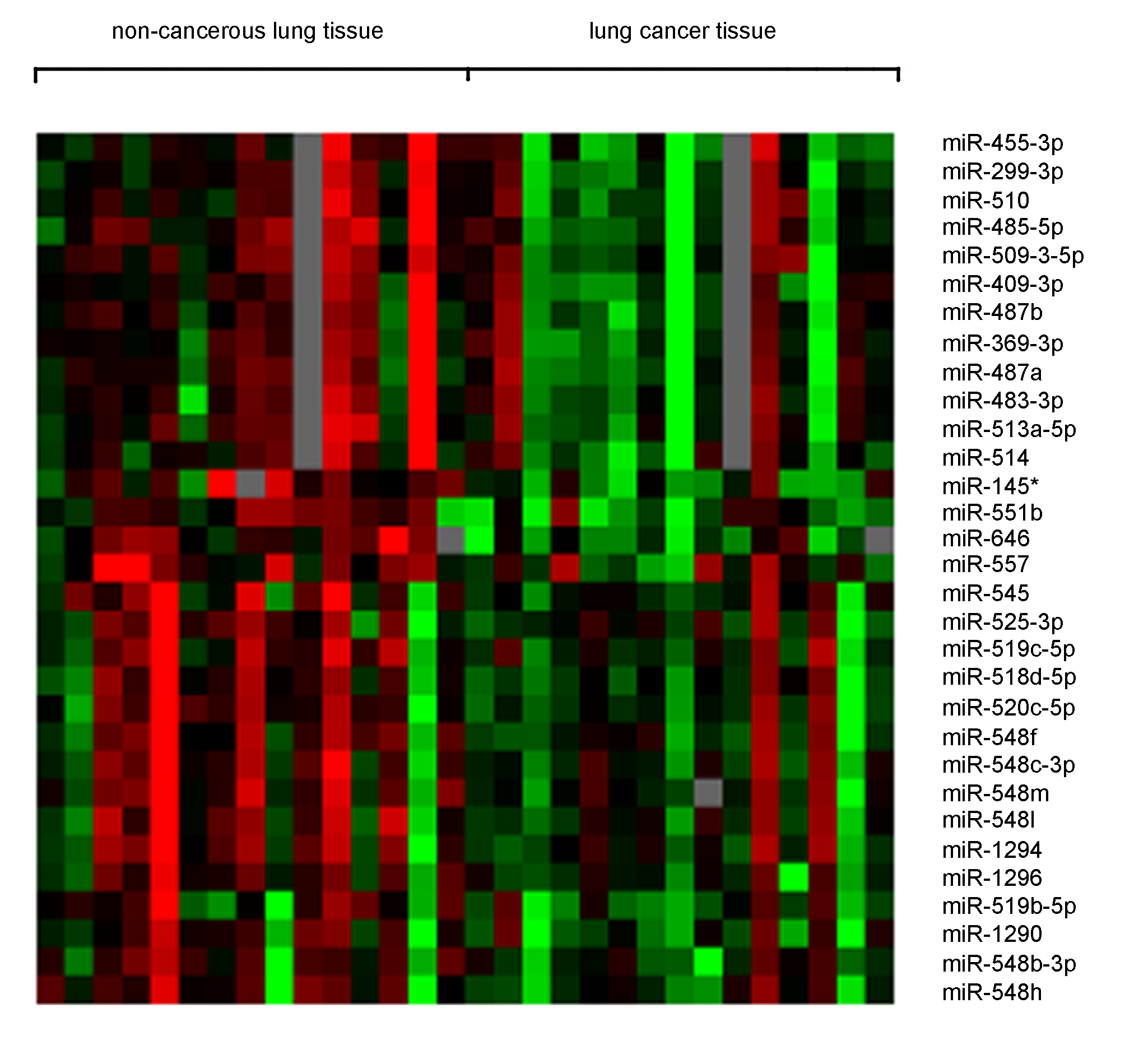

Supplement: Figure S1 — MiRNA expression in lung cancer tissues and adjacent non-cancerous tissues. The expressions of miRNAs are measured with qRT-PCR. 5S rRNA was used as an internal control. The heatmap represents the overexpression (red) and underexpression (green) of miRNAs. Missing data is represented with gray color. (TIF) [file pone.0088022.s001.tif]

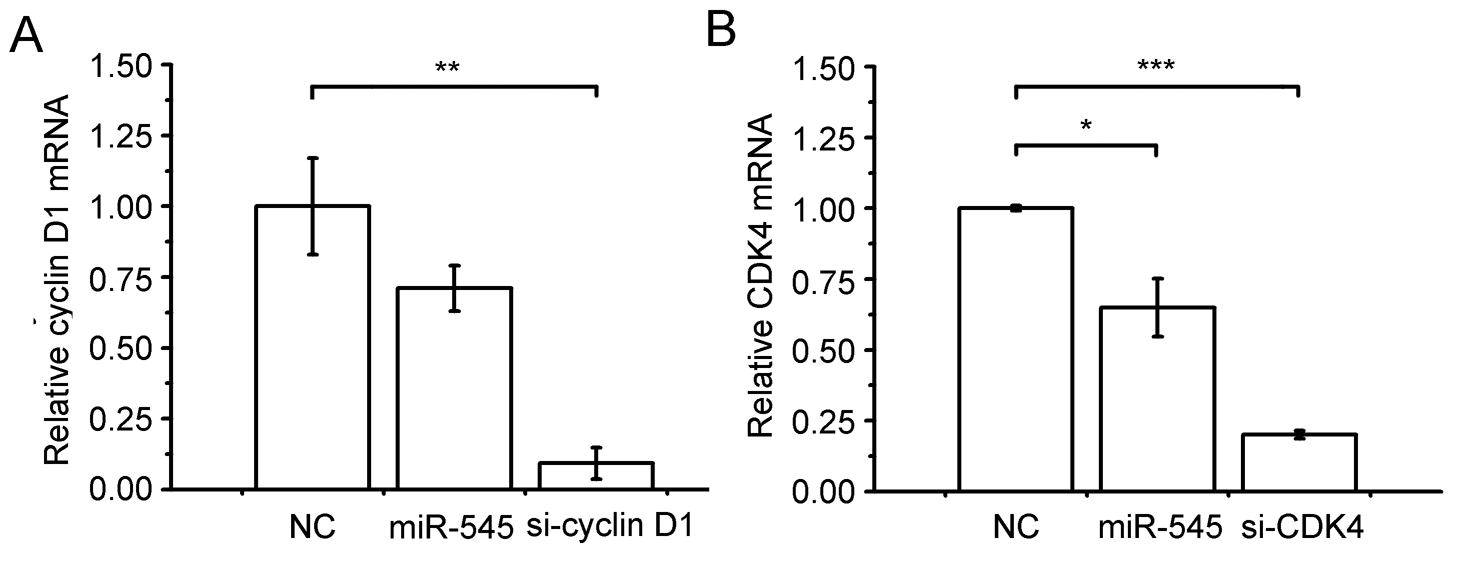

Supplement: Figure S2 — Cyclin D1 and CDK4 mRNAs were measured by qRT-PCR. β-actin was used as an internal control. Data are presented as mean ± S.D. *, P<0.05; **, P<0.01; ***, P<0.001(Student’s t test). (TIF) [file pone.0088022.s002.tif]
