# Supplementary material for: MicroRNA-545 Suppresses Cell Proliferation by Targeting Cyclin D1 and CDK4 in Lung Cancer Cells
Source: PLoS One. 2014 Feb 5;9(2):e88022. doi: 10.1371/journal.pone.0088022 (PMC3914893; doi:10.1371/journal.pone.0088022)
Supplement: Table S1 — The expression of miRNAs in lung cancer tissue and non-cancerous lung tissues. (DOC) [file pone.0088022.s003.doc]

**Table S1. The expression of miRNAs in lung cancer tissue and non-cancerous lung tissues.**

| **miRNAs** | **Fold change (cancer tissue/non-cancerous tissue)** | ***P* value^†^** |
| --- | --- | --- |
| **miR-455-3p** | 0.39 | 0.030 |
| **miR-299-3p** | 0.42 | 0.011 |
| **miR-510** | 0.43 | 0.026 |
| **miR-485-5p** | 0.36 | 0.006 |
| **miR-509-3-5p** | 0.44 | 0.013 |
| **miR-409-3p** | 0.40 | 0.019 |
| **miR-487b** | 0.41 | 0.033 |
| **miR-369-3p** | 0.41 | 0.041 |
| **miR-487a** | 0.45 | 0.041 |
| **miR-483-3p** | 0.48 | 0.048 |
| **miR-513a-5p** | 0.41 | 0.013 |
| **miR-514** | 0.43 | 0.041 |
| **miR-145*** | 0.08 | 0.015 |
| **miR-551b** | 0.31 | 0.015 |
| **miR-646** | 0.11 | 0.002 |
| **miR-557** | 0.30 | 0.031 |
| **miR-545** | 0.17 | 0.036 |
| **miR-525-3p** | 0.13 | 0.036 |
| **miR-519c-5p** | 0.12 | 0.008 |
| **miR-518d-5p** | 0.13 | 0.027 |
| **miR-520c-5p** | 0.16 | 0.011 |
| **miR-548f** | 0.14 | 0.041 |
| **miR-548c-3p** | 0.15 | 0.047 |
| **miR-548m** | 0.09 | 0.017 |
| **miR-548l** | 0.15 | 0.023 |
| **miR-1294** | 0.13 | 0.036 |
| **miR-1296** | 0.16 | 0.012 |
| **miR-519b-5p** | 0.15 | 0.047 |
| **miR-1290** | 0.29 | 0.047 |
| **miR-548b-3p** | 0.16 | 0.027 |
| **miR-548h** | 0.21 | 0.041 |

^†^Wilcoxon test is used to calculate P value.
