# Supplementary material for: MicroRNA-545 Suppresses Cell Proliferation by Targeting Cyclin D1 and CDK4 in Lung Cancer Cells
Source: PLoS One. 2014 Feb 5;9(2):e88022. doi: 10.1371/journal.pone.0088022 (PMC3914893; doi:10.1371/journal.pone.0088022)
Supplement: Table S3 — The characteristics of clinical lung cancer patients. (DOC) [file pone.0088022.s005.doc]

**Table S3. The characteristics of clinical lung cancer** patients.

| **Case No.** | **Age (year)** | **Gender** | **T stage** | **Histological stage** |
| --- | --- | --- | --- | --- |
| 1 | 41 | M | T2 N1 M0 | Ⅱb |
| 2 | 64 | M | T2 N2 M0 | Ⅲa |
| 3 | 61 | M | T3 N0 M0 | Ⅱb |
| 4 | 42 | F | T1 N0 M0 | Ⅰa |
| 5 | 61 | M | T2 N1 M0 | Ⅱb |
| 6 | 62 | M | T1 N1 M0 | Ⅱb |
| 7 | 45 | M | T1 N0 M0 | Ⅰa |
| 8 | 50 | F | T1 N0 M0 | Ⅰa |
| 9 | 74 | F | T2 N0 M0 | Ⅰb |
| 10 | 66 | M | T2 N0 M0 | Ⅰb |
| 11 | 70 | M | T2 N1 M0 | Ⅱb |
| 12 | 58 | F | T1 N0 M0 | Ⅰa |
| 13 | 60 | M | T1 N0 M0 | Ⅰa |
| 14 | 75 | M | T1 N1 M0 | Ⅱa |
| 15 | 77 | M | T2 N0 M0 | Ⅰb |
| 16 | 54 | M | T1 N0 M0 | Ⅰa |
| 17 | 49 | M | T2 N2 M0 | Ⅲa |
| 18 | 65 | F | T2 N2 M0 | Ⅲa |
| 19 | 59 | F | T1 N2 M0 | Ⅲa |
| 20 | 53 | F | T2 N2 M0 | Ⅲa |
| 21 | 67 | F | T1 N2 M0 | Ⅲa |
| 22 | 68 | M | T3 N2 M0 | Ⅲa |
| 23 | 69 | M | T2 N2 M0 | Ⅲa |
| 24 | 54 | F | T2 N2 M0 | Ⅲa |
| 25 | 61 | M | T2a N0 M1b | Ⅳ |
